# Security and efficiency enhancement of an anonymous three-party password-authenticated key agreement using extended chaotic maps

**DOI:** 10.1371/journal.pone.0203984

**Published:** 2018-10-05

**Authors:** Qi Xie, Yanrong Lu, Xiao Tan, Zhixiong Tang, Bin Hu

**Affiliations:** 1 Key Laboratory of Cryptography and Network Security, Hangzhou Normal University, Hangzhou, China; 2 Tianjin Key Laboratory of Advanced Networking, School of Computer Science and Technology, Tianjin University, Tianjin, China; King Saud University, SAUDI ARABIA

## Abstract

Recently, Lu et al. claimed that Xie et al.’s three-party password-authenticated key agreement protocol (3PAKA) using chaotic maps has three security vulnerabilities; in particular, it cannot resist offline password guessing attack, Bergamo et al.’s attack and impersonation attack, and then they proposed an improved protocol. However, we demonstrate that Lu et al.’s attacks on Xie et al.’s scheme are unworkable, and their improved protocol is insecure against stolen-verifier attack and off-line password guessing attack. Furthermore, we propose a novel scheme with enhanced security and efficiency. We use formal verification tool ProVerif, which is based on pi calculus, to prove security and authentication of our scheme. The efficiency of the proposed scheme is higher than other related schemes.

## 1 Introduction

Nowadays it is very common to use mobile devices to conduct transactions via insecure wireless networks [1–2], therefore, how to design secure, efficient and convenient 3PAKA scheme has become an urgent issue for researchers to solve it. Utilizing the semi-group property of Chebyshev polynomial, many extended chaotic maps based 3PAKA protocols were proposed in recent years. However, most of them suffer from security vulnerabilities and low computational efficiency.

In 1995, Steiner et al. [3] extended two-party password-authenticated key agreement to 3PAKA protocol. However, Ding and Horster [4] and Lin et al. [5] demonstrated that their scheme is vulnerable to undetectable online password guessing attack, and Lin et al. [5] further showed that their protocol suffers from offline password guessing attack. To remedy those weaknesses, they proposed an improved 3PAKA protocol, but the server needs to keep a long-term secret key and the parties have to verify server’s public key beforehand. To improve the efficiency, Lin et al. [6] introduced another 3PAKA protocol without using server’s public key. Unfortunately, Chang and Chang [7] pointed out that their improved scheme needs more rounds of communication, and they proposed an ECC-based 3PAKA scheme with better round efficiency. However, Yoon et al. [8] commented that Chang and Chang’s scheme is still insecure against online password guessing attack, and presented an improvement to overcome the weaknesses. But Lo and Yeh [9] commented that Yoon et al.’s protocol is also insecure against undetectable online password guessing attack. Therefore, they developped a secure 3PAKA protocol to eliminate these flaws. Lee et al. [10] and Lu et al. [11] also introduced two enhanced 3PAKA protocols without using server’s public key. Their protocols require multiple modular exponentiation operations to negotiate a session key. Nevertheless, Guo et al. [12] and Phan et al. [13] demonstrated that Lu et al.’s scheme is susceptible to undetectable online dictionary attack, man-in-the-middle attack, and unknown key-share attack, respectively. Then they proposed a scheme with enhanced security, but it requires for more computational cost. In 2014, based on elliptic curve cryptosystems, Xie et al.’s proposed the first secure 3PAKA protocol with user anonymity [14].

Wang et al. [15] proposed the first 3PAKA protocol using chaotic maps (CM-3PAKA) in 2010. Unfortunately, Yoon et al. [16] claimed that their scheme suffers from illegal message modification attack and some other disadvantages. Then Yoon et al. designed a novel 3PAKA protocol to resolve these problems. However, both schemes are inconvenient to use in practice, because these schemes need a trusted third party to pre-share and protect a different long-term secret key. Lee et al. [17] presented an anonymous CM-3PAKA protocol using timestamp in 2013. Unfortunately, Hu and Zhang [18] demonstrated that Lee et al.’s scheme is susceptible to user anonymity attack and man-in-the-middle attack. Moreover, they presented an improved protocol to overcome the weaknesses. In their protocol, the secret session key is established upon Chebyshe chaotic map, which heads from Chebyshev polynomial has the excellent semi-group property. Xie et al. [19] introduced the first extended CM-3PAKA protocol without using timestamp. Later on, Lee et al. [20] showed that Xie et al.’s protocol might suffer from detectable online password guessing attack. Then they proposed a new CM-3PAKA scheme without using password. Unfortunately, their scheme is insecure against impersonation attack [21].

In 2014, Farash and Attari [22] designed a CM-3PAKA scheme without using smart card and server’s public key. The advantage of their scheme is that users only use their passwords to authenticate each other and establish the session key, which can reduce computational costs and avoid public key directory management. Unfortunately, Xie et al. [23] demonstrated that Farash and Attari’s protocol is susceptible to offline password guessing attack and impersonation attack. Then, an improved CM-3PAKA protocol with the same advantage is proposed. The improved scheme is suitable for mobile applications.

In 2016, Lu et al. [24] claimed that Xie et al.’s protocol [23] is susceptible to Bergamo et al.’s attack [25], offline password guessing attack and impersonation attack, and then they proposed an improved scheme to solve these security vulnerabilities. Owing to biometric keys have many advantages compared with single keys, many researchers have attempted to combine password and biometrics keys to provide strong security [26–30]. In this paper, we first discusses Lu et al.’s attacks on Xie et al.’s protocol, and then shows the weaknesses of Lu et al.’s improved protocol, after that, a new protocol based on biometrics is proposed to solve their security vulnerabilities.

Our security goals are as follows:

1. User anonymity: The real identity of each user must be protected during authenticated and key agreement stage.
2. Known-key security: The session key is secure even if the current session key is compromised.
3. Resistant to impersonation attack: The legal user must not be masqueraded by the unauthorized entities.
4. Resistant to password guessing attacks: The password of each user is secure even if the leakage of user’s memory.
5. Resistant to replay attack: The improvement should be able to security against the reusage of the transmitted messages.
6. Resistant to privileged-insider attack: The privileged-insider must not be obtained the plaintext password of each user.
7. Resistant to man-in-the-middle attack: The improvement can withstand this attack if it will not be compromised under impersonation attack and replay attack.

The rest of paper is organized as follows. Sections 2 and 3 present brief review of Xie et al.’s protocol, Lu et al.’s attacks on Xie et al.’s protocol and. Then, Sections 4 and 5 present brief review of Lu et al.’s improved protocol and our security analysis on it. After that, an improved protocol is introduced in Section 6. Security analysis and computation comparisons are presented in Sections 7 and 8. Section 9 concludes the paper.

## 2 Review of Xie et al.’s scheme

Xie et al.’s protocol [23] has four phases: system initialization phase, user registration phase, authenticated key agreement phase, and password change phase. The first three phases are as follows.

### 2.1 System initialization

Let *s* be the secret key of the server *S*, *p* be a large prime number, *h*() be a secure one-way hash function, *H*() be a chaotic maps based one-way hash function, and *x*∈*Z*_*p*_, the parameters {*p*, *h*(), *x*, *H*()} are published and *s* is kept secret.

### 2.2 User registration

Let *UID*_*i*_ and *upw*_*i*_ be user *i*’s identity and password. User *i* computes $UPW_{i}=T_{upw_{i}}(x)\mathrm{mod}p$, and sends {*UID*_*i*_,*UPW*_*i*_} to *S* through a secure channel.

When the server *S* receives {*UID*_*i*_,*UPW*_*i*_}, it computes *VUPW*_*i*_ = *h*(*UID*_*i*_,*s*)+*UPW*_*i*_, and stores {*UID*_*i*_,*VUPW*_*i*_} in its database.

### 2.3 Authenticated key agreement

In this phase, both user *A* and user *B* are authenticated and the session key is established.

**Step 1:** User *A* selects a random number *ua*∈[1,*p*+1], and computes *UR*_*A*_ = *T*_*ua*_(*x*)mod *p*, and sends {*UID*_*A*_,*UID*_*B*_,*UR*_*A*_} to *S*.

**Step 2:** When *S* receives {*UID*_*A*_,*UID*_*B*_,*UR*_*A*_}, it chooses *c*,*d*∈[1,*p*+1], computes

$$UPW_{A}=VUPW_{A}-h(UID_{A},s),$$

$$UPW_{B}=VUPW_{B}-h(UID_{B},s),$$

$$SR_{c}=T_{c}(x)-UPW_{A}\mathrm{mod}p,$$

$$SR_{d}=T_{d}(x)-UPW_{B}\mathrm{mod}p.$$

Then it sends {*UID*_*A*_,*SR*_*d*_} to *B*, and sends {*UID*_*B*_,*SR*_*c*_} to *A*.

**Step 3:** On receiving {*UID*_*A*_,*SR*_*d*_}, *B* chooses *ub*∈[1,*p*+1], computes:

$$UR_{B}=T_{ub}(x)\;\mathrm{mod}p,$$

$$BSK_{BS}=T_{ub}(SR_{d}+UPW_{B})=T_{ubd}(x)\;\mathrm{mod}p,$$

$$BSZ_{BS}=H(0,UID_{B},UID_{A},UR_{B},SR_{d},BSK_{BS}).$$

and sends {*UR*_*B*_,*BSZ*_*BS*_} to *S*.

On receiving {*UID*_*B*_,*SR*_*c*_}, *A* computes:

$$ASK_{AS}=T_{ua}(SR_{c}+UPW_{A})=T_{uac}(x)\;\mathrm{mod}p,$$

$$ASZ_{AS}=H(0,UID_{A},UID_{B},UR_{A},SR_{c},ASK_{AS}).$$

Then, *A* sends {*ASZ*_*AS*_} to *S*.

**Step 4:** On receiving {*UR*_*B*_,*BSZ*_*BS*_} from *B* and {*ASZ*_*AS*_} from *A*, *S* computes *BSK*_*SB*_ = *T*_*d*_(*UR*_*B*_) = *T*_*dub*_(*x*)mod *p* and verifies the correctness of *BSZ*_*BS*_ = *H*(0,*UID*_*B*_,*UID*_*A*_,*UR*_*B*_,*SR*_*d*_,*BSK*_*SB*_). If it is not correct, *S* terminates the request. Otherwise, user *B* is authenticated. After that *S* computes *ASK*_*SA*_ = *T*_*c*_(*UR*_*A*_) = *T*_*cua*_(*x*)mod *p*, and verifies the correctness of *ASZ*_*AS*_ = *H*(0,*UID*_*A*_,*UID*_*B*_,*UR*_*A*_,*SR*_*c*_,*ASK*_*SA*_). If it is not correct, *S* rejects the request. Otherwise, user *A* is authenticated.

After that, *S* computes *SZ*_*AB*_ = *H*(1,*UID*_*A*_,*UID*_*B*_,*UR*_*A*_,*UR*_*B*_,*ASK*_*SA*_), *SZ*_*BA*_ = *H*(1,*UID*_*B*_,*UID*_*A*_,*UR*_*B*_,*UR*_*A*_,*BSK*_*SB*_), and responds {*UR*_*B*_,*SZ*_*AB*_} and {*UR*_*A*_,*SZ*_*BA*_} to *A* and *B*, respectively.

**Step 5:** After receiving {*UR*_*B*_,*SZ*_*AB*_}, *A* verifies the correctness of *SZ*_*AB*_ = *H*(1,*UID*_*A*_,*UID*_*B*_,*UR*_*A*_,*UR*_*B*_,*ASK*_*AS*_). If it is not correct, *A* rejects it. Otherwise, *A* computes *KAB* = *T*_*ua*_(*UR*_*B*_) = *T*_*uaub*_(*x*)mod *p* and *SKAB* = *H*(2,*UID*_*A*_,*UID*_*B*_,*UR*_*A*_,*UR*_*B*_,*KAB*) is the session key shared with user *B*.

When *B* receives {*UR*_*A*_,*SZ*_*BA*_}, he verifies the correctness of *SZ*_*BA*_ = *H*(1,*UID*_*B*_,*UID*_*A*_,*UR*_*B*_,*UR*_*A*_,*BSK*_*BS*_). If it is not correct, *B* rejects it. Otherwise, *B* computes *KBA* = *T*_*ub*_(*UR*_*A*_) = *T*_*ubua*_(*x*)mod *p* and *SKBA* = *H*(2,*UID*_*A*_,*UID*_*B*_,*UR*_*A*_,*UR*_*B*_,*KBA*) is the session key shared with user *A*.

## 3 Comments on Lu et al.’s attacks on Xie et al.’s scheme

Lu et al. claimed that Xie et al.’s CM-3PAKA protocol is vulnerable to Bergamo et al.’s attack, off line password guessing attack and impersonation attack. We will show that their claimed three security vulnerabilities are untenable.

### 3.1 Bergamo et al.’s attack

Lu et al. claimed that Xie et al.’s protocol suffers from Bergamo et al.’s attack, because an adversary can get *T*_*ua*_(*x*) and *T*_*ub*_(*x*) from the public network, and can compute

$$ua^{\prime }=\frac{\arccos(T_{ua}(x))+2k\pi }{\arccos(x)}|k\in Z,\;and\;ub^{\prime }=\frac{\arccos(T_{ub}(x))+2k\pi }{\arccos(x)}|k\in Z,$$

such as *T*_*ua*′_(*x*) = *T*_*ua*_(*x*) and *T*_*ub*′_(*x*) = *T*_*ub*_(*x*). Therefore, the adversary can compute *KBA* = *T*_*ubua*_(*x*)mod *p* and the session key *SKAB* = *H*(2,*UID*_*A*_,*UID*_*B*_,*UR*_*A*_,*UR*_*B*_,*KAB*) shared between users *A* and *B*.

However, this attack actually cannot happen. The reason is that Zhang [31] pointed out that Chebyshev polynomials *T*_*a*_(*x*) has semi-group property, where *x*∈[−∞,+∞]. In Xie et al.’s protocol, because *x*∈*Z*_*p*_, *ua*∈[1,*p*+1] and *ub*∈[1,*p*+1], so they use Chebyshev polynomials defined on [−∞,+∞] to design 3PAKA protocol to avoid Bergamo et al.’s attack. That is, Xie et al.’s protocol does not meet the condition of Bergamo et al.’s attack.

### 3.2 Off line password guessing attack and impersonation attack

Lu et al. claimed that Xie et al.’s scheme suffers from offline password guessing attack, because an adversary can select *ua*∈[1,*p*+1], compute *UR*_*A*_ = *T*_*ua*_(*x*)mod *p* and send {*UID*_*A*_,*UID*_*B*_,*UR*_*A*_} to *S*.

When *S* receives {*UID*_*A*_,*UID*_*B*_,*UR*_*A*_} from the adversary, it chooses *c*,*d*∈[1,*p*+1], computes

$$UPW_{A}=VUPW_{A}-h(UID_{A},s),$$

$$UPW_{B}=VUPW_{B}-h(UID_{B},s),$$

$$SR_{c}=T_{c}(x)-UPW_{A}\mathrm{mod}p,$$

$$SR_{d}=T_{d}(x)-UPW_{B}\mathrm{mod}p.$$

Then it sends {*UID*_*B*_,*SR*_*c*_} to the adversary.

The adversary guesses a password *UPW*_*A*_′ and computes *ASK*_*AS*_′ = *T*_*ua*_(*SR*_*c*_ + *UPW*_*A*_′, and checks whether *ASZ*_*AS*_ = *H*(0,*UID*_*A*_,*UID*_*B*_,*UR*_*A*_,*SR*_*c*_,*ASK*_*AS*_′) is correct or not. If so, the guessed *UPW*_*A*_′ is correct.

Unfortunately, this attack cannot work against our scheme. The reason is that the adversary cannot know the correct *ASZ*_*AS*_, and cannot check whether *ASZ*_*AS*_ = *H*(0,*UID*_*A*_,*UID*_*B*_,*UR*_*A*_,*SR*_*c*_,*ASK*_*AS*_′) is correct or not. In Xie et al.’s protocol, *ASZ*_*AS*_ should be generated by user *A*, that is to say, if an adversary impersonates *A*, then he can not compute the correct *ASZ*_*AS*_ because he does not know the correct *ASK*_*AS*_′. If the adversary intercepts and gets {*UID*_*A*_,*UID*_*B*_,*UR*_*A*_} and *ASZ*_*AS*_ generated by *A*, then he cannot compute *ASK*_*AS*_′ without knowing the correct *ua* chosen by t *A*, so he cannot compute *H*(0,*UID*_*A*_,*UID*_*B*_,*UR*_*A*_,*SR*_*c*_,*ASK*_*AS*_′), not to say checking the equation *ASZ*_*AS*_ = *H*(0,*UID*_*A*_,*UID*_*B*_,*UR*_*A*_,*SR*_*c*_,*ASK*_*AS*_′).

Since Lu et al.’s off-line password guessing attack is not correct, so their impersonation attack on Xie et al.’s scheme is also invalid.

## 4 Review of Lu et al.’s protocol

Lu et al.’s improved protocol has the same four phases as that of Xie et al.’s protocol. Since we only discuss the weaknesses of Lu et al.’s protocol, so the password change phase is omitted.

### 4.1 System initialization

Let *p* be a large prime number, *Sk*∈[1,*p*+1] and *T*_*Sk*_(*x*)mod *p* be the private and public keys of the server *S*, where *x*∈*Z*_*p*_. Let *h*_1_() be a secure one-way hash function and *h*() be a chaotic maps based one-way hash function. *S* keeps *Sk* secret and publishes the parameters {*p*,*x*,*h*_1_(),*h*(),*T*_*Sk*_(*x*)mod *p*}.

### 4.2 User registration

User *i* chooses his identity *UID*_*i*_, a random number *r*_*i*_ and password *upw*_*i*_, and computes *VG*_*i*_ = *h*_1_(*upw*_*i*_,*r*_*i*_), and sends {*UID*_*i*_,*VG*_*i*_} to *S* through a private channel.

When the server *S* receives{*UID*_*i*_,*VG*_*i*_} from the user *i*, it computes *VUPW*_*i*_ = *h*_1_(*UID*_*i*_,*Sk*)+*VG*_*i*_, and randomly chooses *d*_*i*_, stores {*d*_*i*_⊕*Sk*,*VUPW*_*i*_} in its database, sends {*d*_*i*_,*VUPW*_*i*_}to user *i* through a private channel. user *i* stores {*r*_*i*_,*d*_*i*_} in his memory.

### 4.3 Authenticated key agreement

In this phase, both *A* and *B* are authenticated and the session key is established.

**Step 1:** User *A* selects *ua*∈[1,*p*+1], computes $KAS=T_{d_{A}}(T_{Sk}(x)),$, *VG*_*A*_ = *h*_1_(*upw*_*A*_,*r*_*A*_), *FV*_*A*_ = *h*(*UID*_*A*_,*UID*_*B*_,*T*_*ua*_(*x*),*VG*_*A*_), *CV*_*A*_ = *E*_*KAS*_(*UID*_*A*_,*UID*_*B*_,*T*_*ua*_(*x*),*FV*_*A*_), and then he sends *CV*_*A*_ to *S*.

**Step 2:** *S* computes *d*_*A*_⊕*Sk*⊕*Sk* = *d*_*A*_, $KAS=T_{Sk}(T_{d_{A}}(x))$, (*UID*_*A*_,*UID*_*B*_,*T*_*ua*_(*x*)*FV*_*A*_) = *D*_*KAS*_(*CV*_*A*_), *VG*_*A*_ = *VUPW*_*A*_−*h*_1_(*UID*_*A*_,*Sk*), and checks whether *FV*_*A*_ = *h*(*UID*_*A*_,*UID*_*B*_,*T*_*ua*_(*x*),*VG*_*A*_) is correct or not. If not correct, reject it. Otherwise, *S* computes *FV*_*B*_ = *h*(*T*_*ua*_(*x*),*UID*_*B*_), *VG*_*B*_ = *VUPW*_*B*_−*h*_1_(*UID*_*B*_,*Sk*), $CV_{B}=E_{VG_{B}}(T_{ua}(x),FV_{B},UID_{A},UID_{B})$, and sends *CV*_*B*_ to *B*.

**Step 3:** User *B* uses *VG*_*B*_ to decrypt *CV*_*B*_ and get (*T*_*ua*_(*x*),*FV*_*B*_,*UID*_*A*_,*UID*_*B*_), then checks the validity of *FV*_*B*_. After that, *B* chooses *ub*∈[1,*p*+1], computes *HV*_*B*_ = *h*(*UID*_*B*_,*T*_*ub*_(*x*)), $PV_{B}=E_{VG_{B}}(T_{ub}(x),HV_{B})$, and sends *PV*_*B*_ to *S*.

**Step 4:** *S* decrypts *PV*_*B*_, gets (*T*_*ub*_(*x*),*HV*_*B*_), and checks if *HV*_*B*_ = *h*(*UID*_*B*_,*T*_*ub*_(*x*)). If not, reject it. Otherwise, *S* chooses *C*1,*C*2∈[1,*p*+1] and computes *ZV*_*AS*_ = *h*(*UID*_*A*_,*UID*_*B*_,*T*_*ub*_(*x*),*T*_*C*1_(*x*)), $KAS=T_{Sk}(T_{d_{A}}(x))$, *RV*_*AS*_ = *E*_*KAS*_(*T*_*C*1_(*x*),*T*_*ub*_(*x*),*UID*_*A*_,*ZV*_*AS*_), *ZV*_*BS*_ = *h*(*UID*_*A*_,*UID*_*B*_,*T*_*ua*_(*x*),*T*_*C*2_(*x*)), *KBS* = *T*_*Sk*_(*T*_*ub*_(*x*)), *RV*_*BS*_ = *E*_*KBS*_(*T*_*C*2_(*x*),*T*_*ua*_(*x*),*UID*_*B*_,*ZV*_*BS*_), and returns *RV*_*AS*_ to *A*, *RV*_*BS*_ to *B*.

**Step 5:** When *A* obtains *RV*_*AS*_, he decrypts *RV*_*AS*_ and gets (*T*_*C*1_(*x*),*T*_*ub*_(*x*),*UID*_*A*_,*ZV*_*AS*_), then verifies whether *ZV*_*AS*_ = *h*(*UID*_*A*_,*UID*_*B*_,*T*_*ub*_(*x*),*T*_*C*1_(*x*)) is correct or not. If yes, *A* computes *SK*_*AB*_ = *T*_*ua*_(*T*_*ub*_(*x*))mod *p*, *VAB* = *h*(*UID*_*A*_,*SK*_*AB*_), and sends *VAB* to *B*.

*B* verifies the validity of *ZV*_*BS*_ = *h*(*UID*_*A*_,*UID*_*B*_,*T*_*ua*_(*x*),*T*_*C*2_(*x*)), and computes *SK*_*AB*_ = *T*_*ub*_(*T*_*ua*_(*x*))mod *p*, *VBA* = *h*(*UID*_*B*_,*SK*_*AB*_), and sends *VBA* to *A*.

**Step 6:** *A* and *B* check the validity of *VBA* and *VAB*, respectively. If the checking holds, *SK*_*AB*_ = *T*_*ua*_(*T*_*ub*_(*x*))mod *p* is the shared session key between *A* and *B*.

## 5 Analysis on Lu et al.’s protocol

In this section, we show that Lu et al.’s claims are not correct.

### 5.1 Off line password guessing attack

In Lu et al.’s protocol, an adversary can get the verification parameters {*r*_*i*_,*d*_*i*_} stored in users’ mobile terminals by side-channel attack [32–34], then he can do offline password guessing attack.

When *S* receives *CV*_*A*_ from *A*, it computes *FV*_*B*_ = *h*(*T*_*ua*_(*x*),*UID*_*B*_), *VG*_*B*_ = *VUPW*_*B*_−*h*_1_(*UID*_*B*_,*Sk*), $CV_{B}=E_{VG_{B}}(T_{ua}(x),FV_{B},UID_{A},UID_{B})$, and sends *CV*_*B*_ to *B*. The adversary intercepts and gets *CV*_*B*_ from public network, guesses user *B*’s password *PWB* and computes *VG*_*B*_′ = *h*_1_(*PWB*,*r*_*B*_), uses *VG*_*B*_′ to decrypt *CV*_*B*_ and get (*T*_*ua*_(*x*)′,*FV*_*B*_′,*UID*_*A*_′,*UID*_*B*_′), then he computes *h*(*T*_*ua*_(*x*)′,*UID*_*B*_′) and checks whether it is equal to *FV*_*B*_′. If yes, the guessed password is correct. Otherwise, the adversary can do it again untill he gets the correct password.

The adversary can obtain user *A*’s password by using the above method. Therefore, Lu et al.’s protocol is vulnerable to offline password guessing attack.

### 5.2 Stolen-verifier attack

In Lu et al.’s protocol, the server needs to store the verifier messages {*d*_*i*_⊕*Sk*,*VUPW*_*i*_} for each user *i*. Obviously, the registered adversary *C* has his/her own {*d*_*C*_,*VUPW*_*C*_}. If he/she obtains the verifier messages {*d*_*i*_⊕*Sk*,*VUPW*_*i*_} from the database of the server, then he/she can launch stolen-verifier attack. That is to say, the adversary can find *d*_*C*_⊕*Sk* from *VUPW*_*C*_, then he/she can compute server’s private key *Sk* = *d*_*C*_⊕*Sk*⊕*d*_*C*_. After this, the adversary can compute each user’s message and launch server impersonation attack.

## 6 Improved scheme

Our improved protocol also has four phases: system initialization, user registration, authenticated key agreement, and password change.

### 6.1 System initialization

The parameters {*Sk*,*p*,*x*,*h*_1_(),*h*(),*T*_*Sk*_(*x*)mod *p*} are the same as that of Lu et al.’s scheme, and let *H*() be biological information hash function.

### 6.2 User registration

User *i* chooses his identity *UID*_*i*_, a random number *r*_*i*_ and password *upw*_*i*_, and computes *VG*_*i*_ = *h*_1_(*upw*_*i*_,*r*_*i*_), and sends {*UID*_*i*_,*VG*_*i*_} to *S* through a private channel.

When the server *S* receives {*UID*_*i*_,*VG*_*i*_} from the user *i*, it computes *VUPW*_*i*_ = *h*_1_(*UID*_*i*_,*Sk*)+*VG*_*i*_, and stores {*UID*_*i*_,*VUPW*_*i*_} in its database, sends *VUPW*_*i*_ to user *i* through a private channel.

User *i* inputs his biometrics *UBIO*_*i*_, and computes *d*_*i*_ = *H*(*UBIO*_*i*_,*upw*_*i*_), *VR*_*i*_ = *H*(*UBIO*_*i*_)⊕*r*_*i*_, stores {*VR*_*i*_,*d*_*i*_} in his memory.

### 6.3 Authenticated key agreement

In this phase, both *A* and *B* are authenticated and the session key is established (Please see Algorithm 1).

**Step 1:** User *A* enters his or her biometrics *UBIO*_*A*_ and *upw*_*A*_, computes *H*(*UBIO*_*A*_,*upw*_*A*_) and checks if it equals to *d*_*A*_. If not, repeat this process. *A* selects *Ua*∈[1,*p*+1], computes *T*_*Ua*_(*x*), *KAS* = *T*_*Ua*_(*T*_*Sk*_(*x*)), *r*_*A*_ = *H*(*UBIO*_*A*_)⊕*VR*_*A*_, *VG*_*A*_ = *h*_1_(*upw*_*A*_,*r*_*A*_), *FV*_*A*_ = *h*(*UID*_*A*_,*UID*_*B*_,*T*_*Ua*_(*x*),*VG*_*A*_), *CV*_*A*_ = *E*_*KAS*_(*UID*_*A*_,*UID*_*B*_,*T*_*Ua*_(*x*),*FV*_*A*_), and then he sends {*CV*_*A*_,*T*_*Ua*_(*x*)} to *S*.

**Table:** 

User *A*	The server *S*	User *B*
$\begin{array}{l} \mathrm{Enter}\;UBIO_{A},upw_{A}\\ \mathrm{check}\;\mathrm{if}\;d_{A}=H(UBIO_{A},upw_{A})\\ \mathrm{selects}\;Ua\in [1,p+1]\\ T_{Ua}(x)\\ KAS=T_{Ua}(T_{Sk}(x))\\ r_{A}=H(UBIO_{A})⊕VR_{A}\\ VG_{A}=h_{1}(upw_{A},r_{A})\\ FV_{A}=h(UID_{A},UID_{B},T_{Ua}(x),VG_{A})\\ CV_{A}=E_{KAS}(UID_{A},UID_{B},T_{Ua}(x),FV_{A})\\ \;\overset{\left\{CV_{A},T_{Ua}(x)\right\}}{\to } \end{array}$		
	$\begin{array}{l} KAS=T_{Sk}(T_{Ua}(x))\\ \left(UID_{A},UID_{B},T_{Ua}(x),FV_{A})=D_{KAS}(CV_{A}\right)\\ VG_{A}=VUPW_{A}-h_{1}(UID_{A},Sk)\\ \mathrm{check}\;\mathrm{if}\;FV_{A}=h(UID_{A},UID_{B},T_{Ua}(x),VG_{A})\\ FV_{B}=h(T_{Ua}(x),UID_{B})\\ VG_{B}=VUPW_{B}-h_{1}(UID_{B},Sk)\\ CV_{B}=E_{VG_{B}}(T_{Ua}(x),FV_{B},UID_{A},UID_{B})\\ \;\overset{\left\{CV_{B}\right\}}{\to } \end{array}$	
		$\begin{array}{cc} \overset{\left\{CV_{B},PV_{B}\right\}}{\leftarrow } & \;\begin{array}{l} \mathrm{Enter}\;UBIO_{B},upw_{B}\\ \mathrm{check}\;\mathrm{if}\;d_{B}=H(UBIO_{B},upw_{B})\\ r_{B}=H(UBIO_{B})⊕VR_{B}\\ VG_{B}=h_{1}(upw_{B},r_{B})\\ \left(T_{Ua}(x),FV_{B},UID_{A},UID_{B})=D_{VG_{B}}(CV_{B}\right)\\ Ub\in [1,p+1]\\ HV_{B}=h(UID_{B},T_{Ub}(x))\\ PV_{B}=E_{VG_{B}}(T_{Ub}(x),HV_{B},CV_{B}) \end{array} \end{array}$
	$\begin{array}{ccc} \overset{\left\{RV_{AS}\right\}}{\leftarrow } & \;\begin{array}{l} \mathrm{Decrypt}\;PV_{B}\;\mathrm{obtain}\;(T_{Ub}(x),HV_{B},CV_{B})\\ \mathrm{if}\;HV_{B}=h(UID_{B},T_{Ub}(x))\\ S1,S2\in [1,p+1]\\ ZV_{AS}=h(UID_{A},UID_{B},T_{Ub}(x),S1)\\ RV_{AS}=E_{KAS}(S1,T_{Ub}(x),UID_{A},ZV_{AS})\\ ZV_{BS}=h(UID_{A},UID_{B},T_{Ua}(x),S2)\\ RV_{BS}=E_{VG_{B}}(S2,T_{Ua}(x),UID_{B},ZV_{BS}) \end{array} & \;\overset{\left\{RV_{BS}\right\}}{\to } \end{array}$	
$\begin{array}{cc} \begin{array}{l} \mathrm{Decrypt}\;RV_{AS}\;\mathrm{get}(S1,T_{Ub}(x),UID_{A},ZV_{AS})\\ \mathrm{check}\;\mathrm{if}\;ZV_{AS}=h(UID_{A},UID_{B},T_{Ub}(x),S1)\\ SKAB=T_{Ua}(T_{Ub}(x))\mathrm{mod}p\\ N_{A}=h(UID_{A},SKAB) \end{array} & \;\overset{N_{A}}{\to } \end{array}$		$\begin{array}{cc} \overset{\left\{N_{B}\right\}}{\leftarrow } & \;\begin{array}{l} \mathrm{Decrypt}\;RV_{BS}\;\mathrm{get}\;(S2,T_{Ua}(x),UID_{B},ZV_{BS})\\ \mathrm{check}\;\mathrm{if}\;ZV_{BS}=h(UID_{A},UID_{B},T_{Ua}(x),S2)\\ SKBA=T_{Ub}(T_{Ua}(x))\mathrm{mod}p\\ N_{B}=h(UID_{B},SKBA) \end{array} \end{array}$
Check if *N*_*B*_ = *h*(*UID*_*B*_,*SKAB*)		Check if *N*_*A*_ = *h*(*UID*_*A*_,*SKBA*)

The session key is *SKAB* = *T*_*Ua*_(*T*_*Ub*_(*x*))mod *p*

Algorithm 1: The proposed 3PAKA protocol

**Step 2:** *S* computes *KAS* = *T*_*Sk*_(*T*_*Ua*_(*x*)), (*UID*_*A*_,*UID*_*B*_,*T*_*Ua*_(*x*),*FV*_*A*_) = *D*_*KAS*_(*CV*_*A*_), *VG*_*A*_ = *VUPW*_*A*_−*h*_1_(*UID*_*A*_,*Sk*), and checks if *FV*_*A*_ = *h*(*UID*_*A*_,*UID*_*B*_,*T*_*Ua*_(*x*),*VG*_*A*_) is correct or not. If not, reject it. Otherwise, *S* computes *FV*_*B*_ = *h*(*T*_*Ua*_(*x*),*UID*_*B*_), *VG*_*B*_ = *VUPW*_*B*_−*h*_1_(*UID*_*B*_,*Sk*), $CV_{B}=E_{VG_{B}}(T_{Ua}(x),FV_{B},UID_{A},UID_{B})$, and sends { *CV*_*B*_} to *B*.

**Step 3:** User *B* enters his or her biometrics *UBIO*_*B*_ and *upw*_*B*_, computes *H*(*UBIO*_*B*_,*upw*_*B*_) and checks if it equals to *d*_*B*_. If not, repeat this process. *B* computes *r*_*B*_ = *H*(*UBIO*_*B*_)⊕*VR*_*B*_, *VG*_*B*_ = *h*_1_(*upw*_*B*_,*r*_*B*_), and uses *VG*_*B*_ to decrypt *CV*_*B*_ and get (*T*_*Ua*_(*x*),*FV*_*B*_,*UID*_*A*_,*UID*_*B*_), then checks the validity of *FV*_*B*_. After that, *B* chooses *Ub*∈[1,*p*+1], computes *HV*_*B*_ = *h*(*UID*_*B*_,*T*_*Ub*_(*x*)), $PV_{B}=E_{VG_{B}}(T_{Ub}(x),HV_{B},CV_{B})$, and sends {*CV*_*B*_,*PV*_*B*_} to *S*.

**Step 4:** After receiving {*CV*_*B*_,*PV*_*B*_}, *S* can know it is the response from *B* according to *CV*_*B*_ and decrypts *PV*_*B*_ to get (*T*_*Ub*_(*x*),*HV*_*B*_,*CV*_*B*_, and checks if *HV*_*B*_ = *h*(*UID*_*B*_,*T*_*Ub*_(*x*)). If not, reject it. Otherwise, *S* chooses *S*1,*S*2∈[1,*p*+1] and computes *ZV*_*AS*_ = *h*(*UID*_*A*_,*UID*_*B*_,*T*_*Ub*_(*x*),*S*1), *RV*_*AS*_ = *E*_*KAS*_(*S*1,*T*_*Ub*_(*x*),*UID*_*A*_,*ZV*_*AS*_), *ZV*_*BS*_ = *h*(*UID*_*A*_,*UID*_*B*_,*T*_*Ua*_(*x*),*S*2), $RV_{BS}=E_{VG_{{}_{B}}}(S2,T_{Ua}(x),UID_{B},ZV_{BS})$, and returns {*RV*_*AS*_} to *A*, {*RV*_*BS*_} to *B*.

**Step 5:** When *A* obtains **{***RV*_*AS*_}, he decrypts *RV*_*AS*_ and gets (*S*1,*T*_*Ub*_(*x*),*UID*_*A*_,*ZV*_*AS*_), then verifies whether *ZV*_*AS*_ = *h*(*UID*_*A*_,*UID*_*B*_,*T*_*Ub*_(*x*),*S*1) is correct or not. If yes, *A* computes *SKAB* = *T*_*Ua*_(*T*_*Ub*_(*x*))mod *p*, *N*_*A*_ = *h*(*UID*_*A*_,*SKAB*), and sends {*N*_*A*_} to *B*.

*B* decrypts *RV*_*BS*_ and gets (*S*2,*T*_*Ua*_(*x*),*UID*_*B*_,*ZV*_*BS*_), and verifies the validity of *ZV*_*BS*_ = *h*(*UID*_*A*_,*UID*_*B*_,*T*_*Ua*_(*x*),*S*2). If yes, he computes *SKBA* = *T*_*Ub*_(*T*_*Ua*_(*x*))mod *p*, *N*_*B*_ = *h*(*UID*_*B*_,*SKBA*), and sends {*N*_*B*_} to *A*.

**Step 6:** *A* and *B* check the validity of *N*_*B*_ and *N*_*A*_, respectively. If the checking holds, *SKAB* = *T*_*Ua*_(*T*_*Ub*_(*x*))mod *p* is the shared session key between *A* and *B*.

### 6.4 Password change phase

Each user can update his password as follows.

**Step 1:** User *i* enters his/her biometrics *UBIO*_*i*_ and *upw*_*i*_, computes and checks whether *H*(*UBIO*_*i*_,*upw*_*i*_) = *d*_*A*_. If not, repeat this process. Otherwise, User *i* enters a new password $upw_{i}^{*}$, chooses *c*∈[1,*p*+1], and computes *T*_*c*_(*x*), *K*_*iS*_ = *T*_*c*_(*T*_*Sk*_(*x*)), *r*_*i*_ = *H*(*UBIO*_*i*_)⊕*VR*_*i*_, *VG*_*i*_ = *h*_1_(*upw*_*i*_,*r*_*i*_), $VG_{i}^{*}=h_{1}(upw_{i}^{*},r_{i})$, *M*_*i*_ = {Password change request}, $F_{i}=h(UID_{i},T_{c}(x),VG_{i},VG_{i}^{*})$, $C_{i}=E_{K_{iS}}(UID_{i},T_{c}(x),VG_{i}^{*},M_{i},F_{i})$, and then he sends {*C*_*i*_,*T*_*c*_(*x*)} to *S*.

**Step 2:** *S* computes *K*_*iS*_ = *T*_*Sk*_(*T*_*c*_(*x*)), $\left(UID_{i},T_{c}(x),VG_{i}^{*},M_{i},F_{i})=D_{K_{iS}}(C_{i}\right)$, *VG*_*i*_ = *VUPW*_*i*_−*h*_1_(*UID*_*i*_,*Sk*), and checks whether $F_{i}=h(UID_{i},T_{c}(x),VG_{i},VG_{i}^{*})$ is correct or not. If not, *S* computes *R*_*S*1_ = {Reject}, $M_{S1}=h1(0,UID_{i},VG_{i},VG_{i}^{*})$, and sends {*R*_*S*1_,*M*_*S*1_} to user *i*. Otherwise, *S* computes *R*_*S*2_ = {Accept}, $M_{S2}=h1(1,UID_{i},VG_{i},VG_{i}^{*})$, $VUPW_{i}^{*}=h_{1}(UID_{i},Sk)⊕VG_{i}^{*}$, updates {*UID*_*i*_,*VUPW*_*i*_} with {*UID*_*i*_, $VUPW_{i}^{*}$}, and sends {*R*_*S*2_,*M*_*S*2_} to user *i*.

**Step 3:** If user *i* receives {*R*_*S*2_,*M*_*S*2_}, he verifies if $M_{S2}=h1(1,UID_{i},VG_{i},VG_{i}^{*})$ holds or not. If yes, user *i* uses the new password $upw_{i}^{*}$ next time. Otherwise, he verifies $M_{S1}=h1(0,UID_{i},VG_{i},VG_{i}^{*})$ and goes back to Step 1.

## 7 Security analysis

We first use formal tool ProVerif [35] based on applied pi calculus [36], to prove that our protocol satisfies mutual authentication and session key security. Then, we use security analysis to demonstrate that the proposed scheme not only provides common security features, but also is secure against various attacks.

### 7.1 Formal verification

The formal proof has three different parts: the declaration part, the process part and the security property part.

The declaration includes the definition of the components used in the protocol, such as communication channels, variables and constants, functions, etc. Two kinds of channels are used in the scheme: private channel used in the user registration phase, and public channel used in the authenticated key exchange phase, we define them as below:

free sch: channel [private].

free cch: channel.

The constants and variables in the scheme are defined as follows:

const Sk: bitstring [private].

const x: bitstring.

const p: bitstring.

const UBIOA: bitstring [private].

const UBIOB: bitstring [private].

free UIDA: bitstring [private].

free UIDB: bitstring [private].

free upwA: bitstring [private].

free upwB: bitstring [private].

free SKAB: bitstring [private].

free SKBA: bitstring [private].

We define functions for the scheme as follow:

fun h(bitstring): bitstring.

fun h1(bitstring): bitstring.

fun H(bitstring): bitstring.

fun add(bitstring, bitstring): bitstring.

fun xor(bitstring, bitstring): bitstring.

fun T(bitstring, bitstring): bitstring.

fun senc(bitstring, bitstring): bitstring.

fun VUPW(bitstring): bitstring [data].

The functions h, h1, H represent chaotic map hash function, one-way hash function, biometric based hash function, respectively. The function T represents the Chebyshev chaotic maps, and the function VUPW appended by [data] represents VUPW_*i*_ in the scheme. The algebraic characteristics of the above functions are modeled as the following reduction and equations:

reduc forall a: bitstring, b: bitstring; sub(add(a, b), b) = a.

equation forall a: bitstring, b: bitstring; xor(a, xor(a, b)) = b.

equation forall a: bitstring, b: bitstring; T(a, T(b, x)) = T(b, T(a, x)).

reduc forall m: bitstring, k: bitstring; sdec(senc(m, k), k) = m.

We defined the following four events to prove the authentication property:

event UserAAuthed(bitstring).

event UserARequest(bitstring).

event UserBAuthed(bitstring).

event UserBResponse(bitstring).

The process models every participant’s actions and defines the scheme as parallel execution of actions. The scheme’s message sequences are described as below, where messages 6 and 7, messages 8 and 9 are sent in parallel.

Registration:

Message 1: User_*i*_ − − > Server: {*UID*_*i*_,*VG*_*i*_}

Message 2: Server − − > User_*i*_: {*VUPW*_*i*_}

Authenticated key agreement:

Message 3: User_*A*_ − − > Server: {*CV*_*A*_,*T*_*Ua*_(*x*)}

Message 4: Server − − > User_*B*_: {*CV*_*B*_}

Message 5: User_*B*_ − − > Server: {*CV*_*B*_,*PV*_*B*_}

Message 6: Server − − > User_*A*_: {*RV*_*AS*_}

Message 7: Server − − > User_*B*_: {*RV*_*BS*_}

Message 8: User_*A*_ − − > User_*B*_: {*N*_*A*_}

Message 9: User_*B*_ − − > User_*A*_: {*N*_*B*_}

User *A*’s actions are divided into two parts. In the registration phase, she sends {*UID*_*A*_,*VG*_*A*_} to the server, then receives {*VUPW*_*A*_} from it. All communications in this phase are carried out over secure channel sch. In authenticated key agreement phase, user *A* sends message 3 to remote server, and wait for message 6 from remote server, after that she computes session key *SKAB* and the authenticate message *N*_*A*_, and sends it to user *B*. This phase can run more than once. User *A* is defined as:

let UserA =

new rA: bitstring;

let VGA = h1((upwA, rA)) in

out(sch, (UIDA, VGA));

in(sch, xVUPWA: bitstring);

let dA = H((UBIOA, upwA)) in

let VRA = xor(H(UBIOA), rA) in

!(

let dA' = H((UBIOA, upwA)) in

if dA' = dA then

new Ua: bitstring;

let TUA = T(Ua, x) in

let KAS = T(Ua, T(Sk, x)) in

let rA = xor(H(UBIOA), VRA) in

let VGA = h1((upwA, rA)) in

let FVA = h((UIDA, UIDB, T(Ua, x), VGA)) in

let CVA = senc((UIDA, UIDB, T(Ua, x), FVA), KAS) in

event UserARequest(UIDA);

out(cch, (CVA, T(Ua, x)));

in(cch, xRVAS: bitstring);

let (xS1: bitstring, xTUB: bitstring, xUIDA: bitstring, xZVAS: bitstring) = sdec(xRVAS, KAS) in

if xZVAS = h((UIDA, UIDB, xTUB, xS1)) then

let SKAB = T(Ua, xTUB) in

let NA = h((UIDA, SKAB)) in

out(cch, NA);

in(cch, xNB: bitstring);

if xNB = h((UIDB, SKAB)) then

event UserBAuthed(UIDB)

).

User *B* is defined as:

let UserB =

new rB: bitstring;

let VGB = h1((upwB, rB)) in

out(sch, (UIDB, VGB));

in(sch, xVUPWB: bitstring);

let dB = H((UBIOB, upwB)) in

let VRB = xor(H(UBIOB), rB) in

!(

let dB' = H((UBIOB, upwB)) in

if dB' = dB then

in(cch, xCVB: bitstring);

let rB = xor(H(UBIOB), VRB) in

let VGB = h1((upwB, rB)) in

let (xTUA: bitstring, xFVB: bitstring, xUIDA: bitstring, xUIDB: bitstring) = sdec(xCVB, VGB) in

if xFVB = h((xTUA, UIDB)) then

new Ub: bitstring;

let HVB = h((UIDB, T(Ub, x))) in

let PVB = senc((T(Ub, x), HVB, xCVB), VGB) in

event UserBResponse(UIDB);

out(cch, (xCVB, PVB));

in(cch, xRVBS: bitstring);

let (xS2: bitstring, xTUA: bitstring, xUIDB: bitstring, xZVBS: bitstring) = sdec(xRVBS, VGB) in

if xZVBS = h((xUIDA, UIDB, xTUA, xS2)) then

let SKBA = T(Ub, xTUA) in

let NB = h((UIDB, SKBA)) in

out(cch, NB);

in(cch, xNA: bitstring);

if xNA = h((xUIDA, SKBA)) then

event UserAAuthed(xUIDA)

).

The remote server includes two components which run in parallel. The first component represents registration request from new users. We define this component as:

let RegS =

in(sch, (sUIDI: bitstring, sVGI: bitstring));

let VUPWI = add(h1((sUIDI, Sk)), sVGI) in

let VUPW(sUIDI) = VUPWI in

out(sch, VUPWI).

The second one represents the registered users’ authentication key agreement request. When the remote server receives message 3, he computes *CV*_*B*_ and sends message 4 to user *B*. After receives message 5 from user *B*, he computes *RV*_*AS*_, *RV*_*BS*_ and sends message 6, 7 respectively to user *A*, user *B*. We define this component as:

let AuthS =

in(cch, (sCVA: bitstring, sTUA: bitstring));

let sKAS = T(Sk, sTUA) in

let (sUIDA: bitstring, sUIDB: bitstring, sTUA': bitstring, sFVA: bitstring) = sdec(sCVA, sKAS) in

let sVGA = sub(VUPW(sUIDA), h1((sUIDA, Sk))) in

if sFVA = h((sUIDA, sUIDB, sTUA', sVGA)) then

let FVB = h((sTUA', sUIDB)) in

let sVGB = sub(VUPW(sUIDB), h1((sUIDB, Sk))) in

let CVB = senc((sTUA', FVB, sUIDA, sUIDB), sVGB) in

out(cch, CVB);

in(cch, (sCVB: bitstring, sPVB: bitstring));

let (sTUB: bitstring, sHVB: bitstring, sCVB': bitstring) = sdec(sPVB, sVGB) in

if sHVB = h((sUIDB, sTUB)) then

new S1: bitstring;

new S2: bitstring;

let ZVAS = h((sUIDA, sUIDB, sTUB, S1)) in

let RVAS = senc((S1, sTUB, sUIDA, ZVAS), sKAS) in

let ZVBS = h((sUIDA, sUIDB, sTUA', S2)) in

let RVBS = senc((S2, sTUA', sUIDB, ZVBS), sVGB) in

out(cch, RVAS);

out(cch, RVBS).

The Server is defined as a parallel execution of its two components:

let Server =

(!(RegS)|!(AuthS)).

The protocol is the parallel execution of the above parts:

process !UserA|!UserB|Server

The third part formalizes the security property, in particular, it defines the queries that the ProVerif tool will validate. ProVerif verifies the security attributes by checking assertions according to the query statements. It verifies session key security by checking the attacker query. The session key security verification code is shown below. where attacker(SKAB) means that the attacker can eavesdrop or calculate user *A*’s session keySKAB.

query attacker(SKAB).

query attacker(SKBA).

Proverif verifies the authentication attribute by checking the corresponding assertion of the event. An event is an indicator used specifically for authentication validation in Proverif. In the formal model, the authentications processes are modeled as two relations: one relation for user *A* to authenticate user *B* and another for user *B* to authenticate user *A*. The formal relations are defined as:

query id: bitstring; inj-event(UserAAuthed(id)) = = > inj-event(UserARequest(id)).

query id: bitstring; inj-event(UserBAuthed(id)) = = > inj-event(UserBResponse(id)).

**Fig 1:**
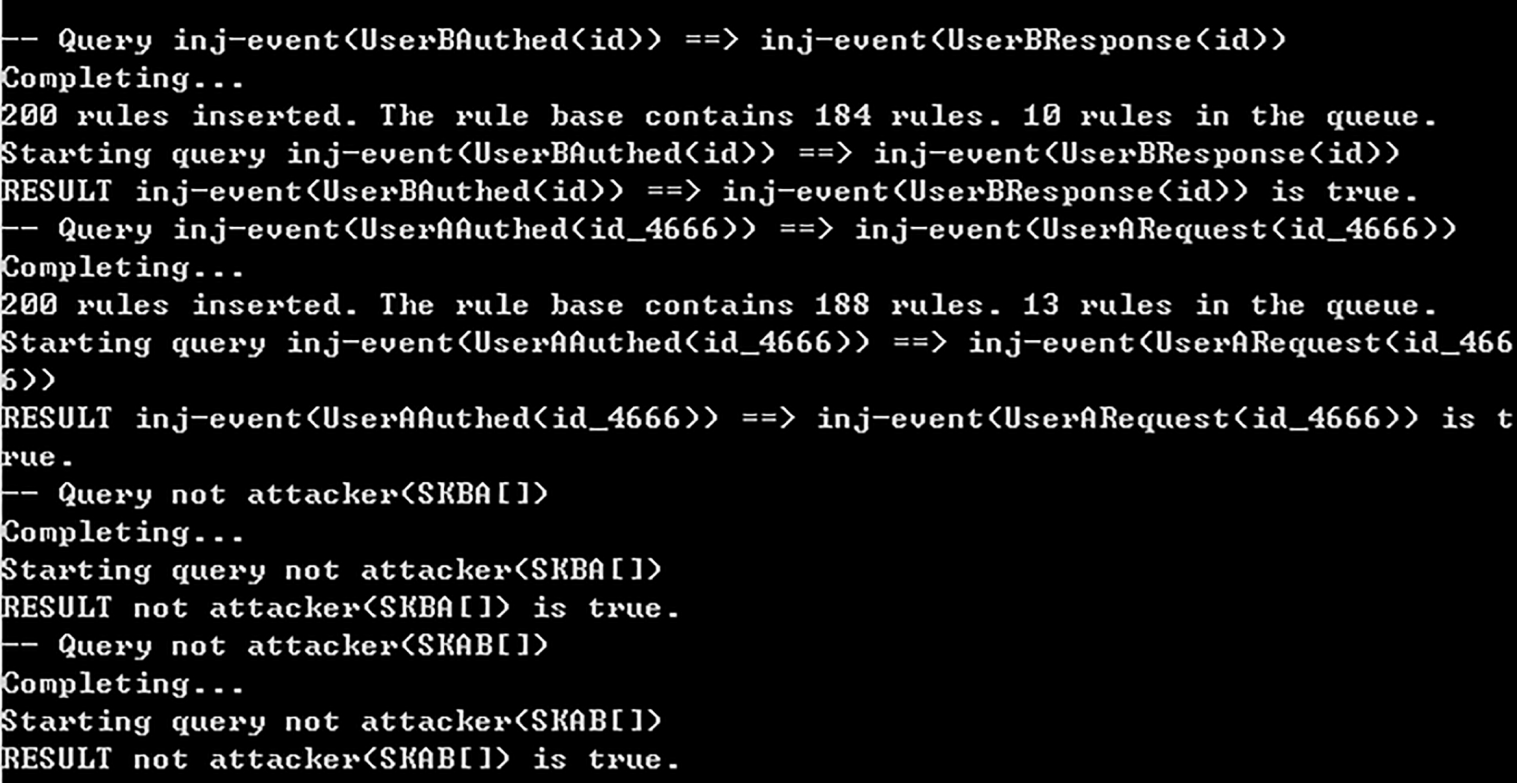
Verification result of the protocol’s authentication and key security property.

We perform the above process in the ProVerif version 1.95. Fig 1 demonstrates that the correspondence queries are true, and the attacker queries are not true. The first result implies that the authentication attribute is satisfied in the presented protocol. The latter result means that the attackers can’t gain the session key, therefore the session key is safe.

### 7.2 Informal analysis

In this section, we discuss that the proposed protocol can resist various known attacks.

#### 7.2.1 User anonymity

In the proposed scheme, the users’ identities are are protected by symmetric cryptographic algorithms and hash functions. Therefore, the adversary can not obtain users’ identities without knowing the secret keys. So the proposed protocol can provide user anonymity.

#### 7.2.2 Password guessing attacks

In the proposed protocol, the users’ passwords are contained in *VG*_*i*_ = *h*_1_(*upw*_*i*_,*r*_*i*_), and *r*_*i*_ = *H*(*UBIO*_*i*_)⊕*VR*_*i*_, where *r*_*i*_ is protected by users’ biometrics *UBIO*_*i*_, therefore, even if an adversary can obtains {*VR*_*i*_,*d*_*i*_} stored in users’ memory, he/she still can not get users’ passwords.

#### 7.2.3 Known-key security

In our scheme, the session key *SKAB* = *T*_*Ua*_(*T*_*Ub*_(*x*))mod *p* depends on two random numbers *Ua* and *Ub*, which varies in different sessions. Thus, the attacker cannot compute previous or future session keys even if he knows the current session key.

#### 7.2.4 Replay attack

Assume the adversary intercepts user *A*’s message {*CV*_*A*_,*T*_*Ua*_(*x*)} and replays it to server. However, upon receiving the message {*RV*_*AS*_}, the adversary cannot decrypt *RV*_*AS*_ and compute the correct message *N*_*A*_ to user *B*, since the attacker cannot compute the decryption key *KAS*, where *KAS* = *T*_*Sk*_(*T*_*Ua*_(*x*)). If the attacker replays user *B*’s message {*PV*_*B*_} to server, as the attacker does not know the random number *Ub*, he cannot compute the decryption key *KBS* = *T*_*ub*_(*T*_*Sk*_(*x*)), when receive the server’s message {*RV*_*BS*_}. If the attacker replays the server’s messages {*CV*_*B*_} to user *B*, he cannot generate the valid message {*RV*_*BS*_}, since he cannot decrypt user *B*’s response message {*PV*_*B*_}.

#### 7.2.5 Privileged-insider attack

In the registration phase of our protocol, user *i* sends {*UID*_*i*_,*VG*_*i*_} to the remote server, where *VG*_*i*_ = *h*_1_(*upw*_*i*_,*r*_*i*_). The privileged-insider attacker cannot guess the user *i*’s password *upw*_*i*_, as it is protected by the random number *r*_*i*_.

#### 7.2.6 Impersonation attack

If the attacker impersonates user *A* or user *B* and sends the message {*CV*_*A*_,*T*_*Ua*_(*x*)} or {*PV*_*B*_,*CV*_*B*_} to the server, he needs to compute the valid *FV*_*A*_ = *h*(*UID*_*A*_,*UID*_*B*_,*T*_*Ua*_(*x*),*VG*_*A*_) or $PV_{B}=E_{VG_{B}}(T_{Ub}(x),HV_{B},CV_{B})$. However, the attacker does not know user *A*’s password *upw*_*A*_ or user *B*’s password *upw*_*B*_, hence, he cannot compute the valid message to pass through the server’s authentication. If the attacker wants to impersonate the remote server, he needs the server’s secret key *Sk*, the verifier messages *VUPW*_*A*_ and *VUPW*_*B*_, which are unaccessable to him.

#### 7.2.7 Man-in-the-middle attack

According to the above analysis, it is impossible for the adversary to launch impersonation attack and replay attack on our protocol. As a result, our protocol can resist the man-in-the-middle attack.

## 8 Security and computation comparisons

Tables 1 and 2 show the security and computational cost comparison between our scheme and some related protocols. For convenience, some notations are used here: let T be the unit time for performing one Chebyshev polynomial computation, E be the unit time for one symmetric encryption/decryption and H be the unit time for one hashing.

**Table 1:** Security comparison between our scheme and other schemes.

Security attributes\Schemes	[20]	[21]	[22]	[23]	[24]	Ours
Biometric keys	N	Y	N	N	N	Y
Provide user anonymity	Y	Y	N	N	Y	Y
Perfect forward secrecy	Y	Y	Y	Y	Y	Y
Known key security	Y	Y	Y	Y	Y	Y
Replay attack	Y	Y	Y	Y	Y	Y
Password guessing attack	Y	Y	N	Y	N	Y
Stolen smart card attack	Y	Y	Y	Y	Y	Y
Privileged-insider attack	Y	Y	Y	Y	Y	Y
Impersonation attack	N	Y	N	Y	N	Y
Stolen-verifier attack	Y	Y	Y	Y	N	Y
Man-in-the-middle attack	Y	Y	Y	Y	Y	Y

If the scheme can prevent the attack or satisfy the property, the symbol ‘Y’ is used. Otherwise, ‘N’ is used.

**Table 2:** Performance comparison of authenticated key agreement.

	User *A*	User *B*	Server *S*	Total	Estimated time
Lee et al. [20]	4T+2E+4H	3T+2E+4H	4T+4E+4H	11T+8E+12H	360.2ms
Xie et al. [21]	3T+2E+7H	3T+2E+7H	2T+4E+6H	8T+8E+20H	265.2ms
Farash et al. [22]	4T+5H	4T+5H	4T+6H	12T+16H	389.6ms
Xie et al. [23]	4T+3H	4T+3H	4T+6H	12T+12H	388.8ms
Lu et al. [24]	3T+2E+5H	3T+3E+6H	5T+5E+7H	11T+10E+18H	362.3ms
Our scheme	3T+2E+5H	2T+3E+7H	1T+5E+7H	6T+10E+19H	201.5ms

Table 1 shows that our protocol owns more secutity properties than other related protocols. According to the protocol proposed by Xue and Hong [37], the actual execution time is as follows: T is about 32.2ms, E is about 0.45ms and H is about 0.2ms. From Table 2, we know that our protocol is more efficient than other related schemes.

## 9 Conclusion

In this paper, we showed that Lu et al.’s attacks on Xie et al.’s scheme are untenable, and further pointed out that their improved protocol is insecure, which suffers from offline password guessing attack and stolen-verifier attack. Therefore, we proposed an improved protocol to eliminate their security vulnerabilities. We showed that our improved protocol possesses user anonymity, known session key security and withstands impersonation attack, reply attack, man-in-the-middle attack, etc. Also, we verified our protocol achieves mutual authentication and the secutity of the session key. Finally, the performance comparison showed that the efficiency of our scheme is higher than other related schemes. In the future, we will apply our protocol to verify its performance in real scenarios.
